# First characterization of the oral microbiota of capybaras (*Hydrochoerus hydrochaeris*) in Brazil: insights into one health risks

**DOI:** 10.1007/s42770-026-01871-6

**Published:** 2026-02-03

**Authors:** Tamires Ataides Silva, Lucianne Cardoso Neves, Warley Vieira de Freitas Paula, Bianca Barbara Fonseca da Silva, Diego Ortiz da Silva, Cíntia Pelegrineti Targueta, Flávia Regina Florencio Athayde, Mariana Pires de Campos Telles, Iveraldo dos Santos Dutra, Felipe da Silva Krawczak, Ana Carolina Borsanelli

**Affiliations:** 1 Setor de Medicina Veterinária Preventiva, Escola de Veterinária e Zootecnia, Universidade Federal de Goiás (UFG), Goiânia, Goiás Brasil; 2 Instituto de Ciências Biológicas, Universidade Federal de Goiás (UFG), Goiânia, Goiás Brasil; 3 Departamento de Produção e Saúde Animal, Faculdade de Medicina Veterinária de Araçatuba, Universidade Estadual Paulista (Unesp), Araçatuba, São Paulo Brasil

**Keywords:** Rodents, Oral microbiota, One health, Bite, Oral health

## Abstract

Capybara (*Hydrochoerus hydrochaeris*), the largest rodent in the world, are animals native to Brazil that are increasingly adapting to urban environments, which raises concerns for One Health, including the risk of zoonotic diseases such as Brazilian Spotted Fever. Moreover, increased interaction between humans and capybaras can lead to accidents involving injuries and infections. This study aimed to characterize the oral microbiota of healthy capybaras in a peri-urban environment in Goiás state, midwestern region of Brazil. Oral clinical examinations were performed, and subgingival biofilm samples were collected from eight capybaras captured on the campus of the Universidade Federal de Goiás. Sequencing of the V4 region of the 16 S ribosomal RNA gene revealed a predominance of the phyla Firmicutes (48.06%), Proteobacteria (22.07%), Actinobacteriota (11.56%), and Bacteroidota (7.14%). The most abundant genera were *Streptococcus* (36.68%), *Rothia* (6.47%), and *Weissella* (2.02%). Microbial composition varied among individuals, with notably lower diversity in the only juvenile evaluated. This interindividual variability may be influenced by dietary and environmental factors. Genera frequently associated with periodontal disease, such as *Fusobacterium* and *Bacteroides*, were detected at low prevalence. The presence of *Streptococcus*, *Rothia*, and *Klebsiella* suggests the pathogenic potential of these microorganisms for humans and animals, especially in the event of accidents. This study is the first to evaluate the oral microbiota of capybaras and opens an important field of research within the One Health framework. These findings reinforce the importance of microbiological monitoring of synanthropic wildlife for both public health and the oral health of the species.

## Introduction

Capybaras (*Hydrochoerus hydrochaeris*) belong to the order Rodentia and are recognized as the largest rodents in the world [1]. Native to Brazil, this species occurs in several South American countries and inhabits areas near bodies of water, including urbanized environments such as public squares, parks, and reservoirs [1–4]. As semi-aquatic animals, capybaras rely on aquatic habitats for reproductive activities, predator avoidance, and thermoregulation [5].

As hystricomorph rodents, capybaras have elodont teeth, characterized by open roots and continuous growth throughout life, which compensates for wear associated with gnawing and feeding habits [6–8]. They are also monophyodont, possessing only one set of teeth during their lifetime, without deciduous or permanent dentition [6]. Their dental formula is 2 x (I 1/1, PM 1/1, M 3/3), totalling 20 teeth, and their dental anatomy is similar to that of the paca (*Agouti paca*) [7, 8].

Studies involving the coypu (*Myocastor coypus*), another hystricomorph rodent, have shown that sugar-rich diets are associated with dental conditions such as fractures, caries, and periodontal disease [9, 10]. A comparative study of capybaras (*H. hydrochaeris*), coypus (*M. coypus*), agoutis (*Dasyprocta leporina*, *D. fuliginosa*, and *D. punctata*), and pacas (*A. paca*) reported a higher prevalence of periodontal disease in grass-consuming species, such as capybaras and coypus, particularly among adult individuals, suggesting a possible association with age [11]. Despite these findings, studies on wild hystricomorph rodents have primarily focused on dental and cranial anomalies and diet-associated pathological changes, largely based on osteological material. In contrast, information on the oral microbiota of these species remains scarce or absent. Here, we provide baseline data on the oral microbiota of clinically healthy *H. hydrochaeris*, establishing a reference for future studies on periodontal disease in wild hystricomorph rodents.

The adaptability of capybaras to synanthropic environments, driven by abundant resources and low predation pressure, has favoured their presence in urban, peri-urban and agricultural areas, often resulting in economic losses such as crop damage [2, 12, 13]. Within a One Health context, capybaras play an important role as primary hosts of the tick *Amblyomma sculptum*, which amplifies *Rickettsia rickettsii*, the causative agent of Brazilian Spotted Fever [14, 15]. Moreover, interactions between capybaras and humans may lead to accidents, as these animals can attack other animals or even people when threatened, causing serious injuries with a risk of infection [16, 17]. The treatment of wounds resulting from such incidents involves antimicrobial prophylaxis, which should be guided by knowledge of the animal’s oral microbiota. However, until now, the oral microbiota of capybaras had not been characterized [16, 18].

The characterization of the oral microbiota of animals is relevant both for human medicine, particularly in cases involving bites injuries, and for understanding oral health in wildlife species, especially regarding dental and periodontal conditions. In this context, the present study aimed to investigate the composition of the oral microbiota of healthy capybaras inhabiting a peri-urban environment in the Midwestern region of Brazil.

## Materials and methods

### Study area

From July to September 2020, subgingival biofilm samples were collected from capybaras (n = 8) captured on the campus of the Universidade Federal de Goiás – UFG (16° 35’ 42.00” S, 49° 16’ 50.00” W, 718 m above sea level), in Goiânia, Goiás, in the midwestern region of Brazil. The animals inhabited the area of the School Farm of the Veterinary Medicine and Animal Science School at UFG. In this area, capybaras roam freely, having access to native vegetation, pasture areas, and, also, to food provided to the cattle, such as corn silage and concentrates. At the time of the study, it was estimated that at least two distinct capybara groups were present in the region. Due to logistical constraints associated with the capture and handling of free-ranging capybaras, the sample size was limited. Therefore, this study was designed as an exploratory analysis aimed at providing a first characterization of the oral microbiota of this species.

### Oral clinical examination and subgingival biofilm collection

Capybaras were captured as described by Neves et al. [19], and an intraoral clinical examination of the animals was conducted using a lip and tongue retractor, periodontal probe, and flashlight. The results were recorded on a dental chart, and clinical periodontal parameters were assessed. The Williams periodontal probe was used for partial periodontal evaluation of the dental arch, which was introduced parallel to the long axis of the labial and lingual surfaces of the incisor teeth, following the modified Triadan system. Due to limited access, probing of the molar teeth was not performed. After the clinical examination, subgingival dental biofilm samples for microbiological evaluation were collected from the animals. The removal of supragingival bacterial biofilm was performed using sterile gauze, and specimens were collected using paper cones and preserved in ultrapure water at −80 °C until DNA extraction, following the criteria outlined by Borsanelli et al. [20].

### DNA extraction and 16 S rRNA sequencing

DNA extraction from dental samples was performed using the GenElute Mammalian Genomic DNA Miniprep Kit (Sigma, St. Louis, United States). Negative controls consisting of blank extractions were included and processed alongside the samples to monitor potential contamination. PCR amplicon libraries targeting the V4 region of the 16 S rRNA gene (515 F-806R) were generated using a primer set with barcodes adapted for the Illumina platforms [21, 22]. The amplicons were paired-end sequenced on an Illumina MiSeq using custom primers and sequencing procedures [22] at the Environmental Sample Preparation and Sequencing Facility (ESPSF) at Argonne National Laboratory, Lemont, United States.

### Sequencing data analysis

The sequencing data from the V4 region of the 16 S rRNA gene were processed according to the DADA2 microbiome pipeline with default parameters [23]. Forward (R1) and reverse (R2) reads were trimmed to 150 bp (truncLen = c(150, 150)), an expected maximum error threshold of 2 was applied to both reads (maxEE = c(2, 2)), and sequences containing ambiguous bases were discarded (maxN = 0). Bacterial communities were identified by unique amplicon sequence variants (ASVs) among the reads, and taxonomy was assigned to each ASV through taxonomic training using the SILVA database (v138) [24]. All analyses were conducted in the R 4.1.2 environment [25], using the R packages DADA2 v.1.22 and DECIPHER v.2.22 [23].

### Statistical analyses

All analyses were performed in R [25] using *tidyverse* for data handling, *phyloseq* for microbiome data management, *vegan* for diversity and β-diversity analyses, and iNEXT for Hill number calculations [26–29].

The ASV table was rarefied, and relative abundances were summarized at the Phylum and Genus levels. Alpha diversity was assessed with the Shannon–Wiener and Simpson indices, and Pielou’s evenness (J) was calculated as H′/ln(S). Hill numbers (q = 0, 1, 2) were used to represent richness, exponential Shannon diversity, and inverse Simpson diversity, respectively.

Beta diversity was calculated with the Bray–Curtis distance based on relative abundances. Community patterns were explored with a heatmap of the distance matrix and a non-metric multidimensional scaling (NMDS) ordination. Compositional differences were tested using PERMANOVA with 999 permutations and Weight, Sex, and Category as predictors. The assumption of homogeneity of multivariate dispersions was tested using the function betadisper in the *vegan* package [27]. Differences in within-group dispersions were assessed with permutest (999 permutations) and pairwise comparisons were further evaluated with Tukey’s HSD when applicable.

## Results

### Examined animals

Clinical examination revealed no macroscopic evidence of periodontal disease, fractures, or other dental abnormalities in any of the examined individuals. The dataset included individuals of both sexes and different age categories, comprising one cub, three juveniles, and four adults (Table 1). Age class classification (cub and juvenile) was based on body weight, as detailed in Table 1, and not on dental characteristics. This distribution reflects the age and sex structure commonly observed in free-ranging capybara groups in urban environments [19].

**Table 1:** Individual information of the capybaras (*Hydrochoerus hydrochaeris*) examined, including identification code, body weight (kg), sex [male (M), female (F) and dominant male (MD)] and age category (cub, juvenile or adult), collected in a peri-urban environment of Brazil

Code	Weight (kgs)	Sex	Category
CAP1	30.7	M	Juvenile
CAP2	27.4	M	Juvenile
CAP3	6.8	F	Cub
CAP4	27.45	F	Juvenile
CAP5	74.1	F	Adult
CAP6	74.5	F	Adult
CAP7	65.3	MD	Adult
CAP8	67	F	Adult

### Sequencing output

Sequencing yielded a total of 514,952 reads for the 8 samples, with a mean of 64,369 sequences per sample (range: 44,192–87,570). After removing sequencing errors and unwanted sequences, 16.56% of the reads were discarded, resulting in a final total of 450,287 sequences. Each sample contained an average of 56,286 sequences (range: 40,227–78,871), and the data were randomly subsampled to 40,227 sequences per sample for downstream analyses. This rarefaction depth was chosen because it corresponded to the lowest sequencing depth among samples, ensuring that alpha and beta diversity metrics were calculated using an equal sampling effort across all samples while minimizing biases related to library size differences. This cutoff still retained approximately 89% of the mean sequencing depth per sample.

**Fig. 1:**
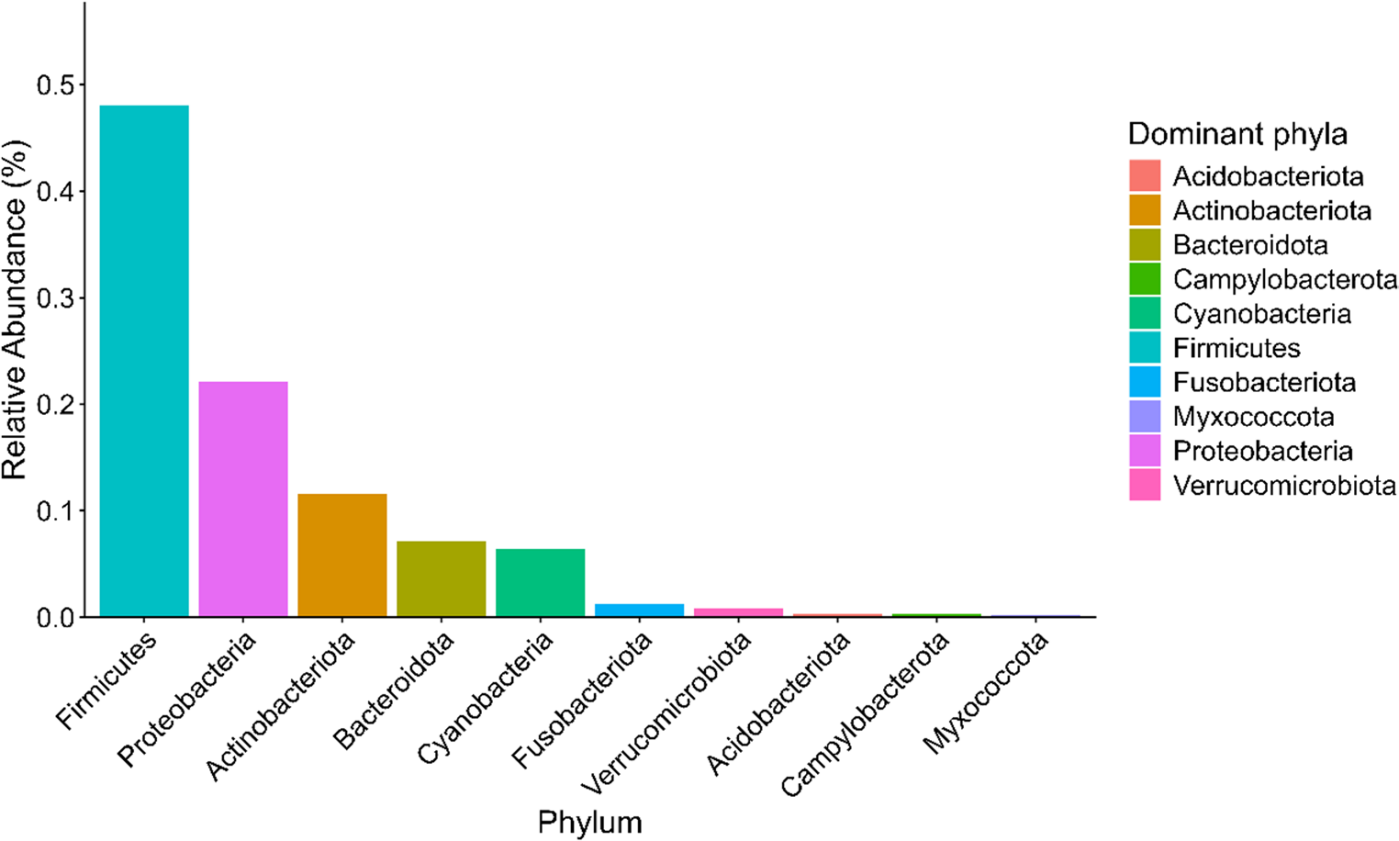
Relative abundance of bacterial phyla identified in the oral microbiota of clinically healthy capybaras (*Hydrochoerus hydrochaeris*) from a peri-urban area of Brazil

A total of 1,346 ASVs were identified across the eight samples. Thirty-five different phyla were detected, of which eight had a relative abundance greater than 0.3%, collectively accounting for 97.68% of the sequences. The most prevalent phyla were Firmicutes (48.06%), Proteobacteria (22.07%), Actinobacteriota (11.56%), Bacteroidota (7.14%), Cyanobacteria (6.38%), Fusobacteriota (1.29%), Verrucomicrobiota (0.84%), and Campylobacterota (0.34%). The distribution of each phylum across the samples is shown in Fig. 1.

**Fig. 2:**
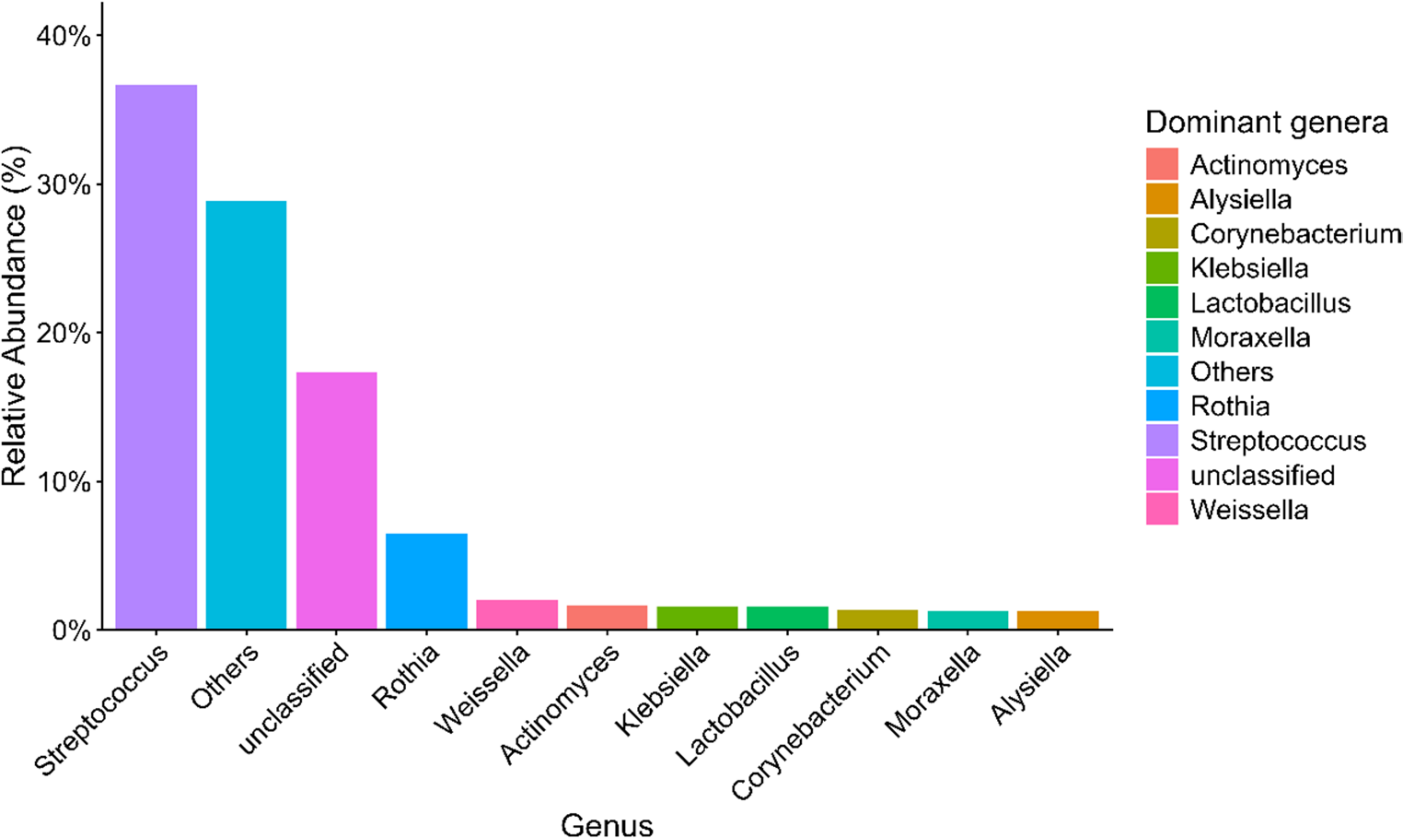
Relative abundance of the most prevalent bacterial genera identified in the oral microbiota of clinically healthy capybaras (*Hydrochoerus hydrochaeris*) from a peri-urban area of Brazil

The ten most prevalent genera across the eight samples were *Streptococcus* (36.68%), *Rothia* (6.47%), *Weissella* (2.02%), *Actinomyces* (1.65%), *Klebsiella* (1.56%), *Lactobacillus* (1.55%), *Corynebacterium* (1.37%), *Moraxella* (1.26%), *Alysiella* (1.26%), and *Bacteroides* (1.23%). The most dominant genera and their relative abundances are shown in Fig. 2, while their reported pathogenic or probiotic roles are summarized in Table 2.

**Table 2:** Main bacterial genera identified in the oral microbiota of clinically healthy capybaras from a peri-urban environment of Brazil and their reported roles

Bacterial genus	Potential role	Reported role
*Rothia*	Pathogen	Opportunistic pathogen associated with bacteremia and other infections in immunocompromised humans; isolated from animal bite wounds [31–34].
*Streptococcus*	Pathogen	Opportunistic oral commensal associated with bite wound infections in humans and reported in periodontal disease in various animal species [16–18,34–36,38].
*Klebsiella*	Pathogen	Opportunistic pathogen reported in human bite wound infections and associated with clinically relevant infections in medical and veterinary contexts [16–18,34].
*Actinomyces*	Pathogen	Oral commensal with opportunistic pathogenic potential; reported in human bite wound infections with clinical relevance [34].
*Corynebacterium*	Pathogen	*C. kutscheri* reported in human infection following rodent bites [34,35].
*Moraxella*	Pathogen	Identified in bite wound infections with clinical relevance [34].
*Fusobacterium*	Pathogen	Associated with periodontal disease in animals [38].
*Bacteroides*	Pathogen	Associated with periodontal disease in animals [38].
*Lactobacillus*	Probiotic-associated	Bacteria with probiotic potential in the oral cavity; contributes to microbial ecological balance and inhibition of periodontal pathogens [42,43].
*Weisella*	Probiotic-associated	Probiotic-associated genus with antimicrobial and immunomodulatory activity against periodontal pathogens [43,44].

The relative abundance analysis of the most representative genera in each sample revealed notable differences in microbial composition. In sample CAP1 (juvenile animal), *Streptococcus* (18.6%) was the dominant genus, followed by *Weissella* (7.4%), with smaller contributions from *Lactobacillus* (6.4%) and *Klebsiella* (3%). CAP2 (juvenile animal) showed a more diverse composition, with *Corynebacterium* (10.7%) as the most abundant genus, followed by *Actinomyces* (8.4%), *Moraxella* (6.2%), and a considerable presence of *Streptococcus* (4.9%). In CAP3 (cub animal), *Streptococcus* (82.9%) was the overwhelmingly dominant genus. Sample CAP4 (juvenile animal) exhibited a distinct microbial profile, with *Streptococcus* (23.5%) as the most represented genus, followed by *Actinomyces* (6.6%) and lower proportions of *Lautropia* (3.4%), *Fusobacterium* (2.5%), and *Bacteroides* (2%).

**Fig. 3:**
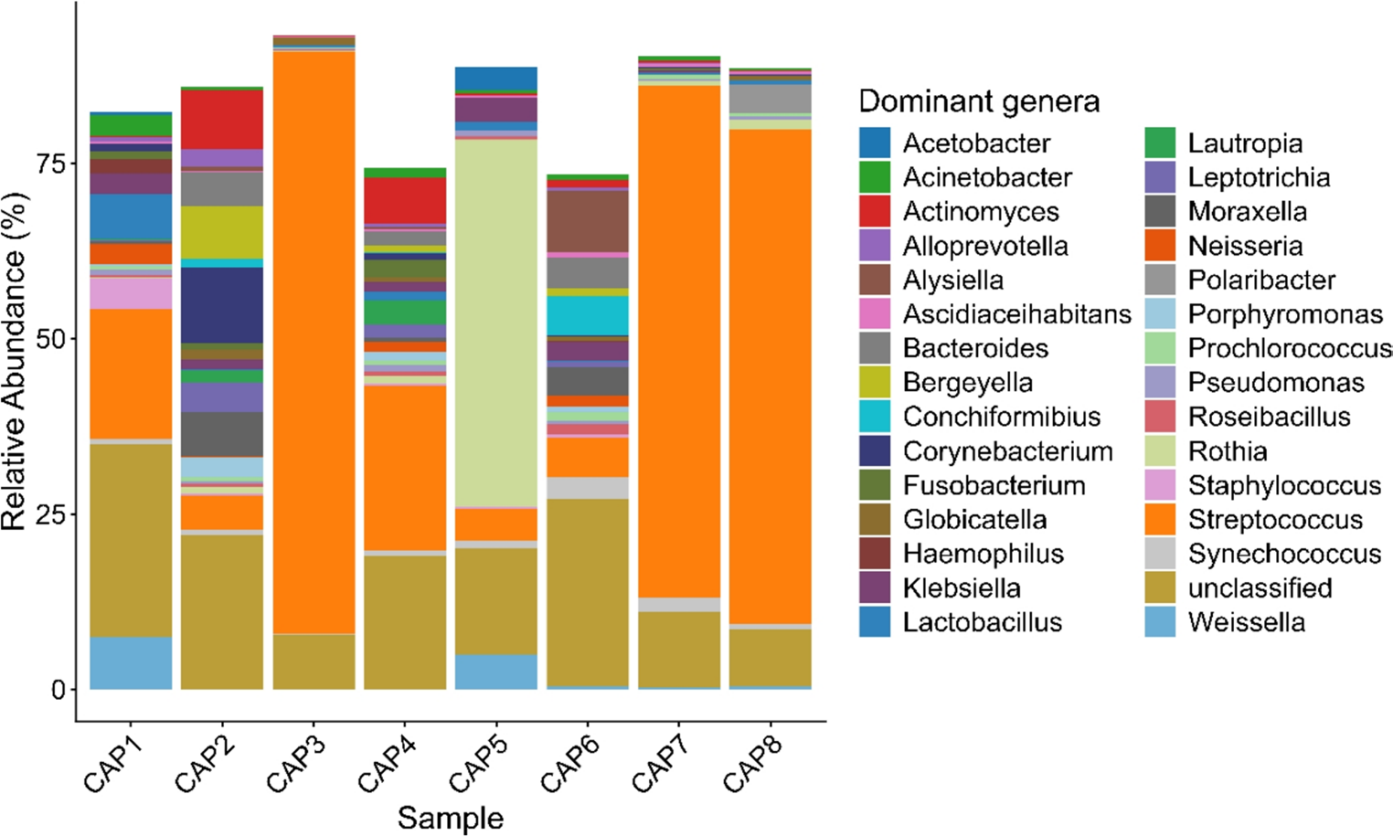
Relative abundance of the 30 most abundant bacterial genera in the oral cavity of clinically healthy capybaras (*H. hydrochaeris*), shown by individual, from a peri-urban environment in Brazil

In sample CAP5 (adult animal), *Rothia* (52.3%) was the predominant genus, with smaller contributions from *Weissella* (4.9%), *Streptococcus* (4.5%), and *Klebsiella* (3.5%). Sample CAP6 (adult animal) showed a more balanced microbial profile, with *Alysiella* (8.7%), *Streptococcus* (5.6%), *Bacteroides* (4.4%), *Moraxella* (4%), and *Klebsiella* (2.6%) being the most represented genera. In samples CAP7 (adult animal) and CAP8 (adult animal), *Streptococcus* was overwhelmingly dominant (73% and 70.4%, respectively), while other genera, such as *Rothia*, were present in lower abundance. These differences in composition indicate significant interindividual variation, with some genera, such as *Streptococcus* and *Klebsiella*, appearing recurrently across samples, while others, including *Actinomyces*, *Weissella*, *Moraxella*, *Bacteroides*, and *Rothia*, showed a more restricted distribution (Fig. 3).

**Fig. 4:**
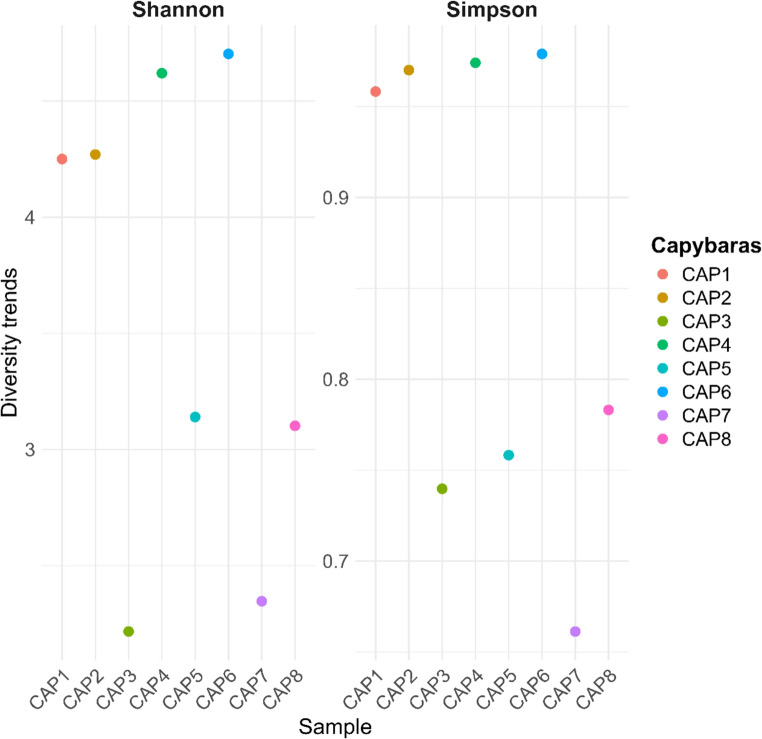
Alpha diversity of the oral microbiota of clinically healthy capybaras (*H. hydrochaeris*) from a peri-urban environment, as estimated by Shannon and Simpson indices. Each point represents an individual sample

Alpha diversity analysis among the samples revealed significant variations in microbial composition (Fig. 4). The Shannon index, which considers both richness and evenness of genera, showed higher values in samples CAP1, CAP2, CAP4, and CAP6, suggesting a more balanced and diverse community in these cases. In contrast, samples such as CAP3 and CAP7 exhibited lower Shannon values, indicating reduced diversity. The Simpson index, which is more sensitive to the dominance of specific genera, supported these observations: CAP1, CAP2, CAP4, and CAP6 also displayed higher values, reflecting high diversity without excessive dominance by a single genus. Conversely, CAP3, CAP5, CAP7, and CAP8 showed relatively low Simpson values, indicating communities dominated by a few genera.

**Fig. 5:**
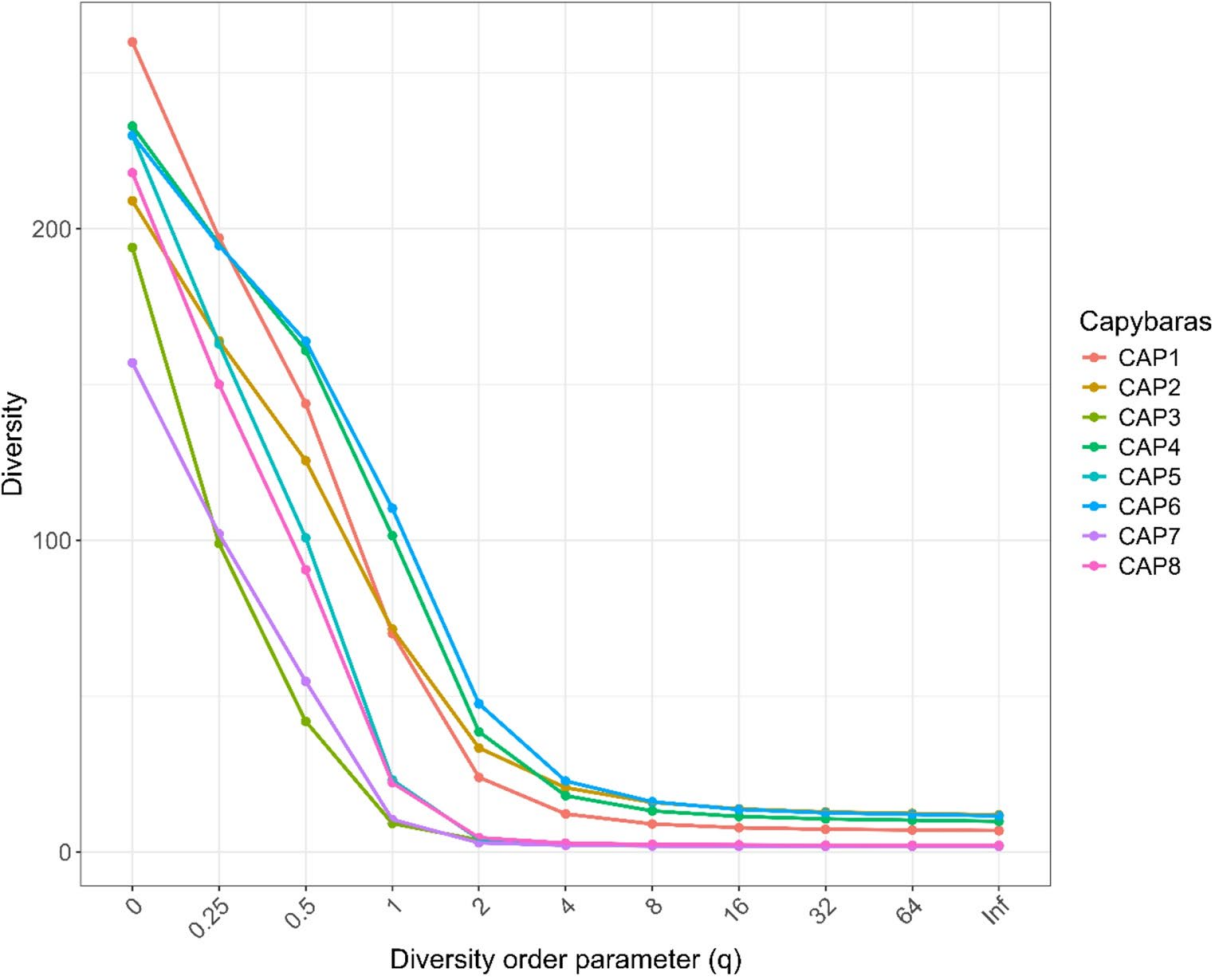
Hill diversity curves illustrating the structure of the oral microbial communities of clinically healthy capybaras (*Hydrochoerus hydrochaeris*) from a peri-urban environment

Hill diversity analyses revealed variations in the structure of microbial communities among the samples. At the beginning of the diversity curve, some samples, such as CAP1 and CAP6, showed higher values, indicating greater diversity when all genera are considered equally, regardless of their abundances. However, as the analysis gives more weight to dominant genera, a decrease in diversity is observed across all samples, suggesting that a few genera predominate in the microbial community of each sample. This downward trend indicates that although genus richness is high, evenness is low—that is, the community is dominated by specific genera. These results demonstrate a heterogeneous microbial structure among individuals, with varying levels of diversity and dominance of specific genera in each sample (Fig. 5).

**Fig. 6:**
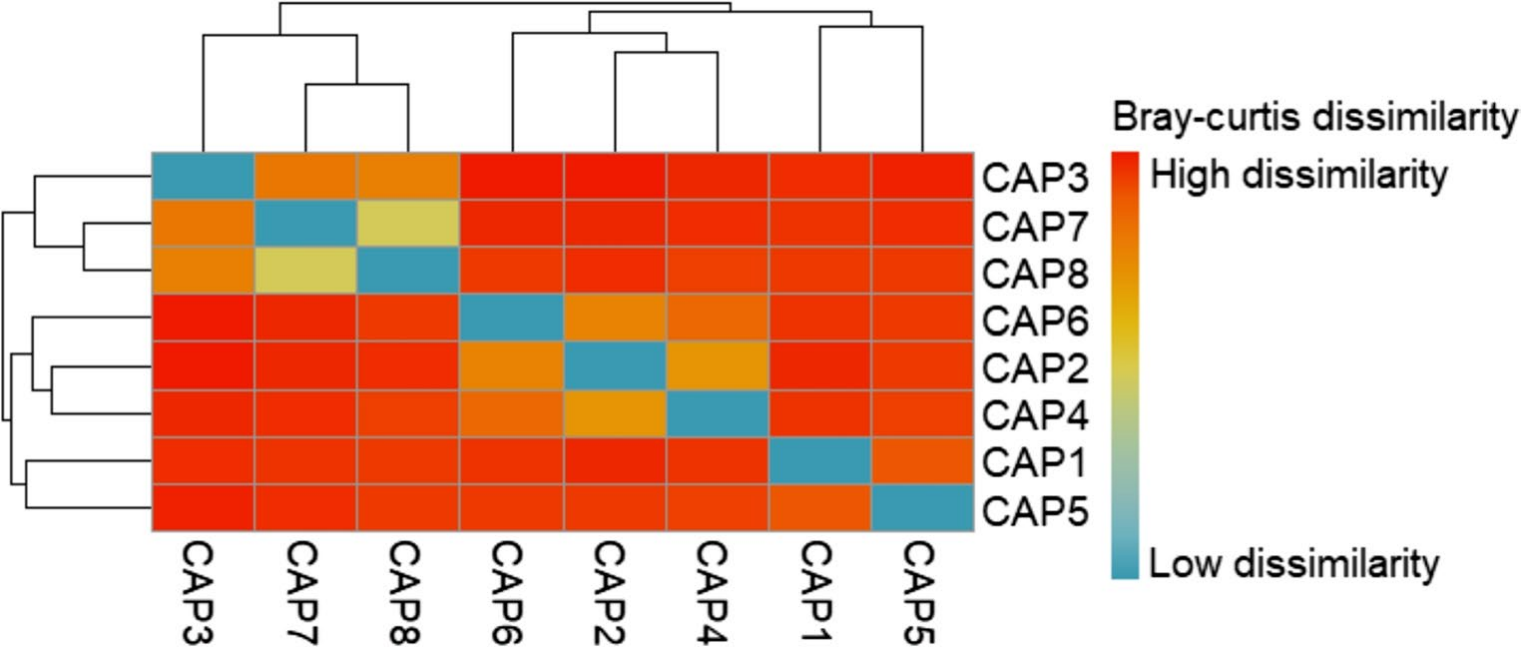
Heatmap showing the similarity between capybara oral microbiota samples based on overall taxonomic composition. Colors represent Bray-Curtis distances, with shades closer to blue indicating greater similarity between samples

The hierarchical clustering heatmaps, based on the overall Bray-Curtis distance matrix, revealed differences in the taxonomic composition of the samples (Fig. 6). Samples CAP7 and CAP8 exhibited similar compositions, while other samples displayed greater variability, indicating that some samples share general taxonomic patterns whereas others differ more substantially.

A permutational multivariate analysis of variance (PERMANOVA) based on Bray–Curtis dissimilarities were used to evaluate the influence of biological factors on microbial community composition. Host category explained a modest proportion of the variation in community structure (R² = 0.39) and showed a marginally significant effect (F = 1.580, *p* = 0.049), whereas host weight (R² = 0.13, F = 1.061, *p* = 0.337) and sex (R² = 0.09, F = 0.748, *p* = 0.774) were not significantly associated with microbial composition (Table 3). Tests for multivariate homogeneity of dispersion indicated no significant differences among categories (betadisper; permutest, *p* = 0.102; ANOVA, *p* = 0.119), suggesting that the PERMANOVA results were not driven by unequal within-group dispersion. Pairwise PERMANOVA comparisons among categories revealed no statistically significant differences after correction for multiple testing (adjusted *p* > 0.44 in all comparisons), despite moderate effect sizes (R² ranging from 0.20 to 0.40). Taken together, these results suggest that host category may be associated with subtle shifts in microbial community structure, warranting further investigation with expanded sampling.

**Table 3:** PERMANOVA results assessing the effects of body weight, sex, and age category on microbial community composition (Bray-Curtis distances) in capybaras (*H. hydrochaeris*)

Factor	Df	Sum of Squares	R2	F	*p* value
Weight	1	0.37787	0.13318	1.0614	0.337
Sex	1	0.26636	0.09388	0.7482	0.774
Category	2	1.12501	0.39651	1.5800	0.049*
Residual	3	1.06805	0.37643		
Total	7	2.83730	1.00000		

**Fig. 7:**
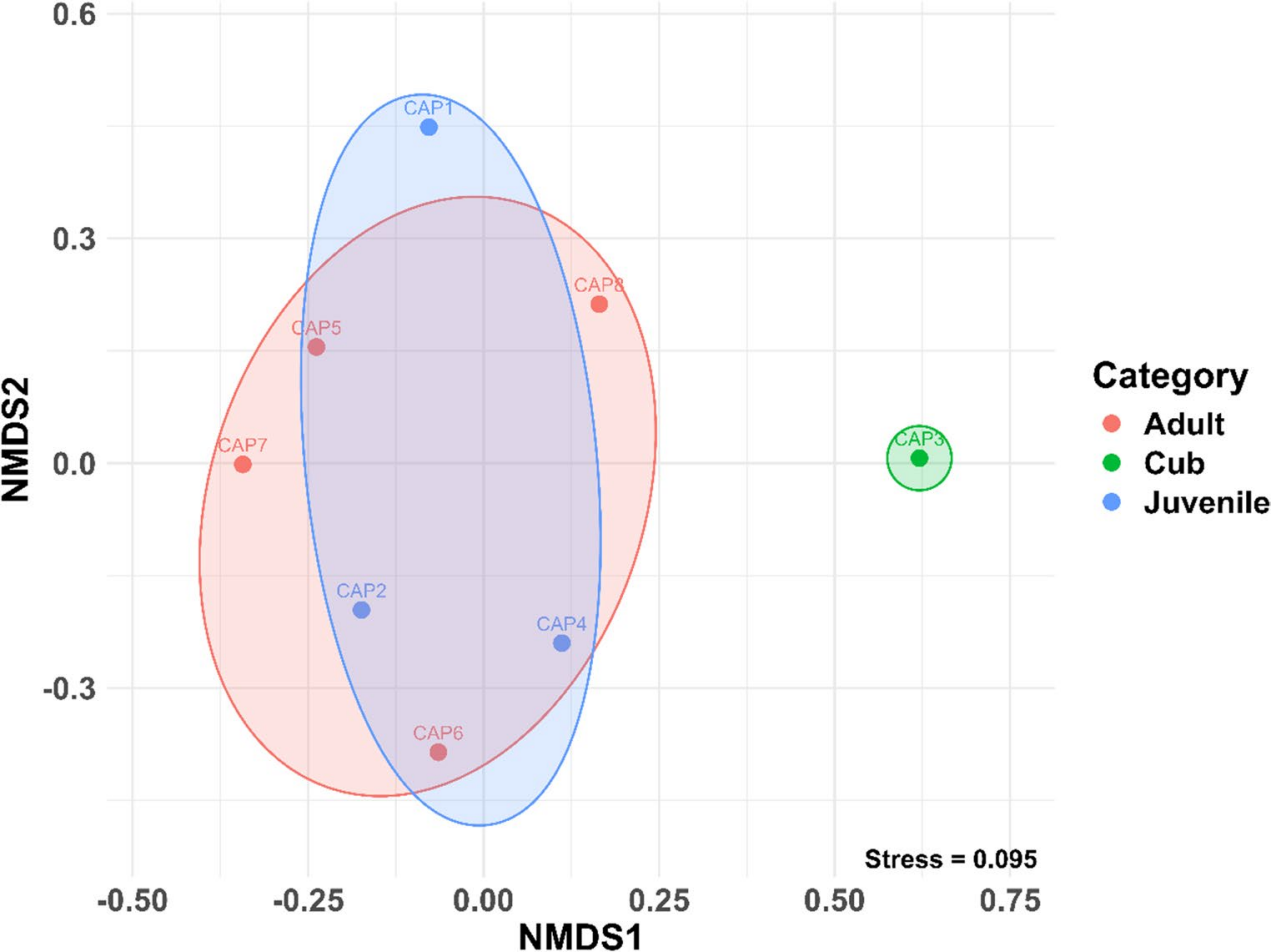
Non-metric multidimensional scaling (NMDS) plot showing differences in microbial community composition among capybara (*H. hydrochaeris*) samples. Each point represents an individual sample, coloured according to its age category or experimental group. Ellipses indicate 95% confidence intervals for each group, highlighting the variation and clustering of microbial communities. Samples that are closer together share more similar bacterial compositions, while those farther apart exhibit more distinct microbial profiles

To visualize these patterns, a non-metric multidimensional scaling (NMDS) analysis was performed. The NMDS plot illustrates the separation of samples based on the similarity of their bacterial communities, where each point represents an individual sample and proximity reflects compositional similarity. Samples positioned closer together share more similar microbial profiles, while those farther apart exhibit distinct community structures, consistent with the PERMANOVA results. These findings indicate that microbial community structure varies among categories, highlighting the importance of group-level factors in shaping microbiota composition (Fig. 7).

## Discussion

Capybaras have successfully adapted to urban, peri-urban and agricultural environments, where their growing population has raised ecological and public health concerns, particularly due to their role as hosts of the tick *Amblyomma sculptum*, the vector of Brazilian Spotted Fever [30]. Given their adaptability to synanthropic environments, characterizing the oral microbiota of this species provides valuable insights into the potential zoonotic implications associated with interactions between capybaras, humans, and domestic animals. Such information is particularly valuable within a One Health framework, as it contributes not only to the assessment of potential zoonotic risks but also to a better understanding of host-microbiota relationships and oral health in a wild rodent species adapted to urban and peri-urban ecosystems.

In the present study, bacterial genera such as *Rothia*, *Streptococcus*, and *Klebsiella* were identified as components of the oral microbiota of capybaras. These genera are of medical and veterinary relevance, often being associated with opportunistic infections in humans and animals. Their presence in the oral cavity of capybaras is particularly relevant given the increasing interactions of this synanthropic species with human populations in urbanized, peri-urban, and rural environments. Additionally, the documented occurrence of bite-related accidents involving capybaras [16–18] highlights the plausibility of microbial transfer during traumatic contact, reinforcing the need to consider the oral cavity of these animals as a potential, albeit indirect, source of exposure to opportunistic microorganisms.

Among the identified microorganisms, *Rothia* deserves particular attention. Although considered part of the normal oral microbiota of humans and animals, species of this genus exhibit opportunistic pathogenic potential and have been associated with infectious conditions in humans, including bacteremia, especially in immunocompromised individuals [31–33]. *Rothia* species have also been isolated from cat bite wounds in humans, even in the absence of overt clinical infection [34], suggesting that their presence alone does not necessarily imply pathogenicity but may represent a latent risk under favorable conditions. Furthermore, *Rothia dentocariosa* has been implicated in the pathogenesis of periodontal disease in humans due to its ability to activate macrophages and modulate host immune responses [37]. In the present study, *Rothia* was detected as part of a diverse microbial community rather than as a dominant taxon, which suggests a low immediate zoonotic risk. Nevertheless, its presence in the oral cavity of capybaras underscores the importance of considering this genus in the context of human-animal interactions involving bites or traumatic contact.

Other bacterial genera identified in this study, including *Streptococcus*, *Actinomyces*,* Klebsiella*, *Lactobacillus*, *Corynebacterium*, and *Moraxella*, have also been reported in wounds resulting from dog bites in humans and have been associated with clinical infections [34]. Notably, *Corynebacterium kutscheri* has been isolated from human infections following rat bites, while *Streptococcus* spp. has been identified in similar incidents without subsequent infectious progression, possibly due to prophylactic antibiotic treatment [35, 36]. Likewise, microorganisms such as *Fusobacterium*, *Streptococcus*, and *Bacteroides*, which were also identified in the present study, have been associated with periodontal diseases in different animal species, including macropods [38]. The identification of these taxa in a synanthropic wild species such as the capybara highligths its potential role as a reservoir of microorganisms shared across wildlife, domestic animals, and humans, reinforcing the relevance of monitoring microbial communities at the human–animal–environment interface.

A plausible explanation for the absence of periodontal lesions in the assessed animals lies in the distinctive dental physiology of capybaras, particularly their continuous dental growth [6–8]. This characteristic may limit long-term biofilm accumulation and reduce the exposure of periodontal tissues to predisposing factors, thereby decreasing susceptibility to periodontal disease. In addition to elodont dentition, the consumption of grasses and fibrous vegetation promotes continuous physiological dental wear, which contributes to occlusal balance and reduces biofilm accumulation, thus favoring oral health maintenance. Together, these factors likely favor the maintenance of oral health and may explain why periodontal diseases are considered uncommon in exotic rodents and squirrels [39, 40]. However, cases of periodontitis have been reported in rodents, including older capybaras and rats [11, 41].

A second hypothesis for the absence of periodontal lesions in the assessed individuals may be related to the distribution and relative abundance of potentially pathogenic microorganisms. It was observed that only one animal harbored the genus *Fusobacterium*, and only two showed bacteria from the *Bacteroides* phylum, with a low prevalence compared to other microorganisms found. This relative abundance suggests that, although these animals do not yet exhibit periodontal lesions, they may have a predisposition to develop the disease in the future, especially under conditions of oral microbiota imbalance and immune response activation by periodontopathogens. In macropods with periodontitis, the abundance of *Fusobacterium* was 23%, and *Bacteroides* was 14.8%, significantly higher values, which may indicate that, in cases of microbial imbalance or predisposing factors, these microorganisms could become more prevalent and contribute to the development of periodontal disease [38].

On the other hand, some bacterial genera identified in this study have gained attention for their probiotic or protective potential in oral ecosystems, including *Weissella* and *Lactobacillus* [42, 43]. These microorganisms play a role in maintaining ecological balance within the oral cavity by limiting the proliferation of pathogenic taxa. *Weissella* species, for example, can inhibit the production of interleukins induced by periodontal pathogens such as *Fusobacterium nucleatum* in oral epithelial cells, as well as exhibiting antimicrobial activity against these microorganisms in vitro [43, 44]. Similarly, *Lactobacillus* species have been shown to exert antagonistic activity against key periodontal pathogens, including *Streptococcus mutans*, *Porphyromonas gingivalis*, and *Prevotella intermedia*, through strain-specific antimicrobial mechanisms [42]. In this study, animals harboring these species did not present *Fusobacterium* bacteria in their oral microbiota, which may reflect the complexity of microbial interactionsin the oral cavity. From an ecological perspective, the presence of these bacteria may benefit the host by contributing to oral microbial homeostasis, limiting the establishment or overgrowth of periodontopathogens, and potentially reducing the risk of periodontal diseases.

The analysis of alpha diversity revealed marked variation among individuals. The least diverse oral microbiota was observed in the only juvenile sampled, which likely reflects an early stage of microbial colonization. In humans, the oral microbiota during early life is characterized by limited diversity, dominated by genera such as *Streptococcus*, *Rothia*, and *Gemella*, with diversification occurring alongside tooth eruption, dietary changes, and immune maturation [45]. A similar process may occur in capybaras, with adult individuals exhibiting more complex and stable microbial communities that reflect long-term host–microbiota interactions and environmental exposure.

In the present study, a tendency toward clustering by life stage was observed, although substantial inter-individual variability in oral microbiota composition remained evident. Despite all sampled capybaras being clinically healthy and originating from the same geographic area, each individual exhibited a distinct microbial profile, suggesting that factors beyond shared environment and host condition may contribute to community structure. Differences in feeding behavior and diet may partially explain this variability, as some individuals consumed bovine silage available in the surrounding area, while others relied primarily on natural vegetation from the reservoir margins. Dietary heterogeneity has been shown to strongly influence oral microbial communities in rodents [9, 10, 46]. The inclusion of a single cub in the dataset may have further contributed to the observed dispersion and influenced clustering patterns. In addition, individuals may belong to different social groups, which can affect feeding strategies, movement patterns, and social interactions, potentially shaping oral microbiota composition. In this context, the lack of statistically significant pairwise differences, despite moderate effect sizes, likely reflects limited statistical power associated with small and uneven sample sizes rather than the absence of biological differentiation. Future studies incorporating larger and more balanced sample sizes, along with detailed information on diet and social structure, will be essential to more robustly assess life-stage–related patterns and identify shared features of the oral microbiota among individuals.

The results of the present study contribute to the characterization of the oral microbiota of capybaras inhabiting peri-urban environments and provide relevant insights for future research in One Health and wildlife health. As the first study to evaluate the oral microbiota of capybaras, this work advances understanding of microbial communities at the interface between wildlife, humans, and domestic animals. The identification of microorganisms with potential pathogenicity emphasizes the importance of continuous monitoring of synanthropic wildlife, particularly in urban and peri-urban areas where close contact increases the likelihood of microbial exchange. From a clinical perspective, these findings may offer baseline information to support antimicrobial prophylaxis strategies following capybara bite injuries. Moreover, the detection of bacteria associated with periodontal diseases highlights the need for further studies focused on oral health and welfare in capybaras, with implications for disease prevention and population management. Additionally, these findings may serve as a reference for microbiological monitoring programs of synanthropic wildlife populations, aiding in the early detection of potentially pathogenic microorganisms and supporting public health surveillance efforts.

From a One Health perspective, the integration of microbiological data from synanthropic wildlife is essential in urban and peri-urban environments characterized by frequent interactions among humans, domestic animals, and wildlife. Future research should prioritize longitudinal and functional approaches to clarify the role of the oral microbiota in host health, disease prevention, and microbial transmission dynamics across the human-animal-environment interface.

## Data Availability

The datasets presented in this study can be found in online repositories. The names of the repository/repositories and accession number(s) can be found at: https://www.ncbi.nlm.nih.gov/, ID PRJNA1381104.
